# Jean-Ovide Decroly (1871–1932): neurologist and pedagogue

**DOI:** 10.1055/s-0046-1825518

**Published:** 2026-07-14

**Authors:** Sylvain Wagnon, Norberto Dallabrida, Léo Coutinho, Hélio A. Ghizoni Teive

**Affiliations:** 1 Université de Montpellier, Montpellier, France.; 2 Universidade do Estado de Santa Catarina, Florianópolis SC, Brazil.; 3 Universidade Federal do Paraná, Hospital de Clínicas, Departamento de Medicina Interna, Programa de Pós-Graduação em Medicina Interna e Ciências da Saúde, Grupo de Doenças Neurológicas, Curitiba PR, Brazil.; 4 Universidade Federal do Paraná, Hospital de Clínicas, Departamento de Medicina Interna, Serviço de Neurologia, Unidade de Distúrbios do Movimento, Curitiba PR, Brazil.

**Keywords:** Neurology, History of Medicine, Cognition, Intellectual Disability, Teaching

## Abstract

The present article aims to understand Ovide Decroly1s intervention in the pedagogical field considering his medical-neurological training. Thus, it shows that, in 1901, Decroly founded an institute of special education and, 6 years later, created the Hermitage School, for disabled and so-called *normal children* respectively. In these educational endeavors, Decroly used the mental tests of the Binet-Simon scale, but indicating their limits; and in the light of the experience of American psychology, in the 1920s, he developed, with Raymond Buyse, the Decroly-Buyse tests.

## INTRODUCTION

**Figure 1:**
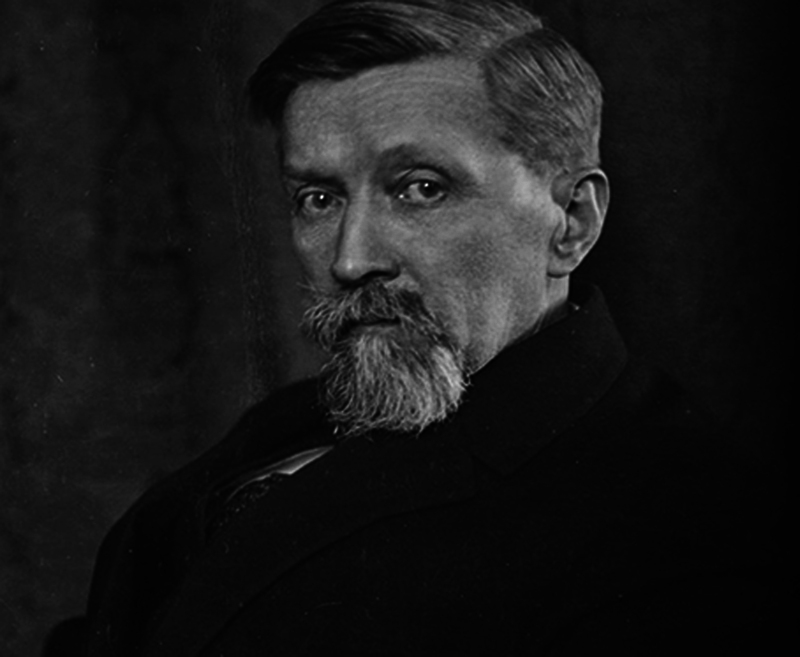
Jean-Ovide Decroly (1871–1932). Extracted from Google Images (Alphabet Inc).

Jean-Ovide Decroly (1871–1932) is best known for the elaboration of the new education pedagogy, based on the ‘centers of interest’ or fundamental needs of children that structure learning, and he is also often referred to as the inventor of the so-called *global* method of writing and reading ( Figure 1 ). 1 2 However, he is, first and foremost, a doctor. His neurological training influenced his educational observations, particularly through the study of mental tests. 3 4 5

This dual approach marks his innovative work in the field of education and support for children with disabilities. In methodological terms, Decroly's trajectory may be read through a Bourdieusian lens, particularly regarding the formation of habitus and the circulation among fields. The transfer of clinical categories to the school context and his critical use of the Binet-Simon scale illustrate how agents mobilize specific forms of capital as they move across the medical, psychological, and educational fields. Such mediation helps explain Decroly's innovative approach to childhood assessment and special education. 6

The present paper discusses Decroly's contributions to the field of medicine, particularly concerning the medical and educational needs of children with intellectual disabilities.

## EARLY YEARS AND NEUROLOGICAL TRAINING

Jean-Ovide Decroly was born in 1871 in the small Belgian town of Renaix. He showed an early liking for the natural sciences, influenced by his father, but also for music and art, influenced by his mother. Decroly detested his two boarding schools and their classical curriculum, with Greek and Latin education, instead of the natural sciences and art he preferred. His admittance to the Medical School of the University of Gent sparked his interest, and he later turned to pathological anatomy of the brain and mental illness. 1 2

His 2 study trips to Berlin in 1896 and his 8-month stay at the Neurology Service of Hôpital Salpêtrière in Paris in 1897, then under the coordination of Fulgence Raymond (1894–1910), were instrumental in his neurological training. 3 7 8 In Berlin and Paris, he observed the institutionalization of psychology and the ‘medicalization’ ★ of social questions at the end of the 19th century in Europe. Back in Belgium in 1898, he joined the neurological department of the Brussels Polyclinic as an assistant to Dr. Zénon Glorieux, one of the pioneers of neurology in Belgium. 9

In 1899, Ovide Decroly became a member of the Belgian Society of Neurology and the Belgian Society of Mental Medicine. He also joined the editorial board of the *Journal de Neurologie* . His numerous writings from 1899 to 1901, particularly in the journal *La Polyclinique* and the *Journal de Neurologie* , mark the continuity of his earlier medical work on nervous diseases and his inclination towards the study of the so-called *irregular* † childhood. 3 4 5 9

## THE SOCIETY FOR THE PROTECTION OF ABNORMAL CHILDREN

In 1901, in Brussels, Ovide Decroly founded a special teaching institute which became the epicenter for all his observations and pedagogical innovations. 4 In the same year, he created the Society for the Protection of Abnormal Children, where he worked actively to classify children whom he called *irregular* and organize special education establishments in Belgium. 5 He worked on anthropometry and psychometry to measure the intelligence of children and detect intellectual delays. His work contributed to the development of a scientific psychopedagogy and the emergence of psychometrics. 5

## THE QUEST FOR AN ADEQUATE INTELLIGENCE TEST: THE CONTRIBUTIONS OF BINET, SOLLIER, AND DECROLY

Ovide Decroly acknowledged the work of French psychologist Alfred Binet to develop a scientific tool to identify children in need of specialized schooling. The Binet-Simon scale, developed by Binet and Théodore Simon at the beginning of 1905, offered standardized tests assessing children's cognitive development, such as memory, attention, and problem-solving skills. 10 11 12 13 An important scientific correspondence was then established between the two men.

Although Ovide Decroly recognized the importance of the Binet-Simon tests for a better understanding of children with disabilities, he also underlined their limitations. 13 He believed that these tests focused too much on cognitive aspects and neglected essential elements of a child's development, such as their family and social and cultural environments, as well as their motor and moral skills. Decroly also warned against the risk of using these tests as rigid criteria for school ranking, thus excluding some children instead of helping them progress in their development. 14

Paul Sollier (1851–1933) is recognized as one of Professor Charcot's most brilliant disciples. 15 He is considered the pioneer of clinical neuropsychology, with several studies on memory. Another area of interest for Sollier was mental retardation, on which he wrote his medical thesis, entitled ‘Psychologie de l’idiot et de l'imbécile.' Sollier's studies preceded those of Binet and Decroly and contributed to the intellectual quotient (IQ) scales. 15 16

Ovide Decroly developed his tests in collaboration with the researcher Raymond Buyse. These tests are inspired by the works of Binet-Simon, but also by those of the Americans Goddard and Terman, professors of psychology at Stanford University, who proposed a revision of the Binet-Simon metric scale with the ‘Stanford-Binet’ test. For Decroly, the main interest of the American tests lay in the creation of standardized, measurable, and transposable tests, defining the aptitudes of each child. 17

The Decroly-Buyse tests, set up in 1925, were intended to detect mental difficulties and promote a more ‘rational’ professional orientation. 16 For Decroly, these tests should enable an evolution of teaching methods thanks to a better knowledge of the children, but also continuous development of the tests throughout schooling. This was to make professional orientation more ‘scientific’ and adequate to the real abilities of the students. 18

In conclusion, while Jean-Ovide Decroly is one of the most influential pedagogues in history, his work in the field of pediatric neuropsychiatry and his quest to create an instrument for intellectual evaluation tailored to the needs of clinicians, pedagogues, and children, are noteworthy.

## Footnotes

★ Medicalization: Historical process through which social or educational issues come to be interpreted and managed according to categories, concepts, and practices derived from the medical field.

## Footnotes

† “Irregular” childhood: Term used in the early twentieth century to designate children considered outside prevailing school and moral norms, including intellectual disabilities, learning difficulties, or behaviors regarded as deviant.
